# Natural antioxidant substances improve oxidative stress and alleviate ulcerative colitis: a meta-analysis of randomized controlled trials

**DOI:** 10.1038/s41598-025-30617-x

**Published:** 2025-11-28

**Authors:** Shuo Yuan, Lingling Cai, Man Su

**Affiliations:** 1 Department of General Surgery, Beilun Branch of the First Affiliated Hospital of Zhejiang University, Ningbo, China; 2 Department of Outpatient Office, Ningbo Mingzhou Hospital, Ningbo, China; 3 Department of Medical Oncology, Beilun Branch of the First Affiliated Hospital of Zhejiang University, Ningbo, China

**Keywords:** Ulcerative colitis, Antioxidants, Oxidation, Oxidative stress, Remission, Diseases, Gastroenterology, Medical research

## Abstract

Oxidative stress is a key driver of mucosal damage in ulcerative colitis (UC). Antioxidant supplementation may restore redox balance, but its clinical efficacy remains controversial. To evaluate the effects of natural antioxidant substances supplementation on oxidation/antioxidant biomarkers and clinical outcomes in UC patients. The databases included PubMed, Embase, Web of Science, and Cochrane Library (up to July 2025) were searched, and RCTs comparing oral antioxidants with placebo in UC were included. The primary outcomes were changes in oxidative stress markers (MDA, SOD, TAC, GPX). The secondary outcomes included the short IBD questionnaire (SIBDQ) score and the simple clinical colitis activity index (SCCAI) score. 9 articles involving 624 patients were included in this study. Compared to placebo group, antioxidant substances supplementation could significantly reduce the level of MDA (*P* = 0.001, SMD=-1.09, 95% CI −1.75 to −0.43), increased the levels of SOD, TAC, GPX in patients with UC (*P* = 0.02, SMD = 0.57, 95% CI 0.10 to 1.04; *P* = 0.0004, SMD = 0.74, 95% CI   0.33 to 1.16; *P* = 0.004, SMD = 0.69, 95% CI  0.22 to 1.16). In addition, antioxidant substances supplementation remarkably decreased the SCCAI score of UC patients (*P* = 0.04, SMD=-0.62, 95% CI −1.21 to −0.04), thus improving the disease activity. However, there is no significant difference in the change of IBDQ score (*P* = 0.13, SMD = 0.55, 95% CI −0.17 to 1.28). Natural antioxidant substances supplementation effectively enhanced antioxidant capacity and ameliorated oxidation status, improved the disease activity of UC patients, but had no significant impact on their quality of life. This study will provide a basis for the selection of adjuvant therapy drugs for UC.

## Introduction

Ulcerative colitis (UC) is a chronic non-specific inflammatory disease, characterized by inflammation of the rectum, colon mucosa, and submucosal lesions^1^. The main clinical manifestations include abdominal pain, diarrhea, mucous purulent stools, and it is difficult to cure and prone to cancer^2^. Globally, the annual number of new cases of ulcerative colitis (UC) is approximately 6.3 per 100,000 person, higher than Crohn’s disease (CD), and the age of onset tends to be younger, which seriously threatens the life and health of Chinese residents^3^. The pathogenesis of UC is not yet clear, but it is closely related to environmental factors, genetic factors, immune tolerance, and intestinal microbiota imbalance^4^. The drug treatment for UC mainly aims to achieve clinical remission and improve the quality of life of patients. At present, clinical treatment drugs for UC mainly include corticosteroids, aminosalicylic acid preparations, immunomodulators and biologic agents^5^. However, long-term use of these drugs may cause drug resistance, and increase the risk of anemia, thrombocytopenia, infection, and tumor formation^6^. Therefore, selecting appropriate plans for the diagnosis and treatment of UC has become an urgent medical issue that needs to be addressed.

Previous studies have found that oxidative stress plays an important role in the pathogenesis and progression of UC^7^. In mammalian cells, oxygen metabolism, as the core of maintaining life activities, inevitably produces some reactive oxygen species (ROS). Under normal physiological conditions, these ROS play a crucial role in maintaining intestinal homeostasis and intestinal health^8^. When the production of ROS exceeds the normal range or the rate of elimination slows down, the resulting imbalance will trigger oxidative stress. When the oxidative stress response is excessive, it can cause the intestinal mucosal layer to be injured, leading to epithelial cell apoptosis. This pathological process weakens the barrier function of the intestine, allowing bacteria to invade the intestine and thereby stimulating the body to produce an immune response^9^. In addition, there is a delicate oxidative antioxidant balance in the intestine, with moderate ROS acting as signaling molecules to participate in immune defense, while antioxidant enzymes such as superoxide dismutase (SOD) and glutathione peroxidase (GPX) promptly clear excess ROS^10^. However, a large number of infiltrating immune cells (such as neutrophils and macrophages) explosively produce ROS in the UC state, causing the ROS levels in colon tissue to soar to 3–5 times higher than those in a healthy state, while the activity of key antioxidant enzymes is significantly reduced^11^. Excessive ROS attacks the lipids, proteins, and DNA of intestinal epithelial cells, leading to cell death and disruption of tight junction structures, resulting in the phenomenon of intestinal leakage^12^.

In recent years, the antioxidant therapy for UC has gradually attracted people’s attention. The purpose of antioxidant therapy is to alleviate the adverse effects of traditional treatments and improve the quality of life of patients. Antioxidants can clear ROS and enhance antioxidant defense, while inhibiting peroxidase, which may be useful in the treatment of UC. Vitamin D participates in the regulation of the immune system and may play a key role in the pathogenesis of IBD. It is considered a promoting factor in IBD treatment, and high levels of vitamin D are associated with low recurrence frequency of IBD. The vitamin D level of UC patients was generally lower than that of healthy individuals, and negatively correlated with the Mayo score^13^. In a double-blind randomized clinical trial, two vitamin D supplementation regimens (1000 or 2000 IU/day) were found to reduce disease activity and improve quality of life in UC patients, but there was no significant change in oxidant/antioxidant status^14^. At present, synthetic antioxidants have been widely used as viable alternatives to natural antioxidants, but their safety has always been controversial. Existing research has confirmed that long-term consumption of synthetic antioxidants can increase the risk of gastrointestinal disorders and cancer susceptibility^15^. Natural antioxidants such as curcumin, resveratrol, quercetin, genistein, etc. have the characteristics of multi-target, high efficacy, and minimal side effects, and have become important research objects for the treatment of ulcerative colitis^10^. Natural antioxidants can play a therapeutic and preventive role in UC by inhibiting inflammatory reactions, regulating the immunity, reducing oxidative stress, improving the intestinal mucosal barrier, and regulating the gut microbiota^16^.

Currently, a number of clinical, randomized and controlled studies have evaluated the therapeutic benefits of natural antioxidants for UC. A randomized, double-blind, placebo-controlled clinical trial investigated the effects of resveratrol supplementation (500 mg/day) on inflammatory biomarkers and quality of life in UC patients^17^. The results showed that resveratrol can reduce oxidative stress, improve antioxidant capacity, improve the quality of life of UC patients, and reduce recurrence rates. Another study evaluated the therapeutic effect of the mediterranean diet (MD) combined with resveratrol and curcumin on UC. The results showed that the MD, MD + curcumin, and MD + resveratrol could effectively reduce disease activity and inflammation in UC patients, improve their quality of life, but there was no statistical difference between the groups^18^. Another clinical study has shown that although direct oral administration of natural antioxidants (such as curcumin) is effective in animal models, their efficacy significantly fluctuates in human trials, which is due to the differences in intestinal absorption capacity and heterogeneity of the lesion microenvironment among patients^19^. Therefore, this study conducted a meta-analysis to comprehensively evaluate the effects of natural antioxidants on oxidative stress status and remission rates in patients with UC.

## Methods

### Search strategy

This meta-analysis was conducted following the Preferred Reporting Items for Systematic Reviews and Meta-analyses (PRISMA) guidelines, and registered in PROSPERO (CRD420251129515). The search databases include PubMed, Web of Science, Cochrane Library, and Embase, and the search period is from the establishment of the database to July 31, 2025. The search terms include: “ulcerative colitis” or “UC” and “antioxidants” or “natural antioxidants” or “natural products” and “oxidative stress”, and the types of studies were restricted to randomized controlled trials (RCTs).

### Inclusion and exclusion criteria

Inclusion criteria: ① all patients were diagnosed with ulcerative colitis and treated with natural antioxidants supplementation; ② The literature type is RCTs and have been published; ③ all included studies have complete basic and interest data. Exclusion criteria: ① Non-RCTs; ② Repeatedly published literature; ③ Literature without clear diagnostic methods and outcome indicators; ④ Due to various reasons, it is not possible to obtain the full text of the literature.

### Data extraction

Two authors (S Yuan and M Su) independently screened the literature and extracted data based on inclusion and exclusion criteria. If there is any disagreement after summarizing, the two researchers will discuss and reach a consensus; if necessary, the third author will determine. The general information and outcomes of all studies were extracted using an excel table. The general information included the first author, publication time, type of literature, sample size, age of patients, disease course, intervention measures, dosage, and treatment course. The primary outcomes were the changes in malondialdehyde (MDA), superoxide dismutase (SOD), total antioxidant capacity (TAC), and glutathione peroxidase (GPX). The secondary outcomes included the short IBD questionnaire (SIBDQ) score and the simple clinical colitis activity index (SCCAI) score.

### Quality assessment

The quality of included literature was evaluated using the Cochrane Collaboration bias risk assessment tool from seven aspects, including randomization, allocation concealment, blinding of participants and personnel, blinding of outcome assessment, outcome data integrity, selective reporting, and other biases. Each item can be judged as low risk, high risk, or unclear risk. If there are any differences after summarizing, they will be discussed or determined by the third researcher.

### Statistical analysis

All the data analysis was conducted using RevMan5.4 software. The quantitative data are represented as mean and standardized mean difference (SMD), while binary variables are represented by odds ratio (OR), both of which are represented by effect values and 95% confidence intervals (CIs). The chi-square test was used for heterogeneity, and the values of *P* and *I*^*2*^ were employed to determine the magnitude of heterogeneity. If *P* ≥ 0.05 and *I*^*2*^ ≤ 50%, there is no statistically significant difference in heterogeneity among the studies, and a fixed effects model is used for analysis. If *P* ≤ 0.05 and *I*^*2*^ > 50%, the heterogeneity is significant, and a random effects model is used. In addition, sensitivity analysis is used for heterogeneity analysis. If heterogeneity still exists after eliminating it, descriptive analysis is used. The funnel plot for the outcome indicators was used to analyze whether there is publication bias. The test level for meta-analysis is set to α = 0.05.

## Results

### Characteristics and quality evaluation of the included studies

**Fig. 1:**
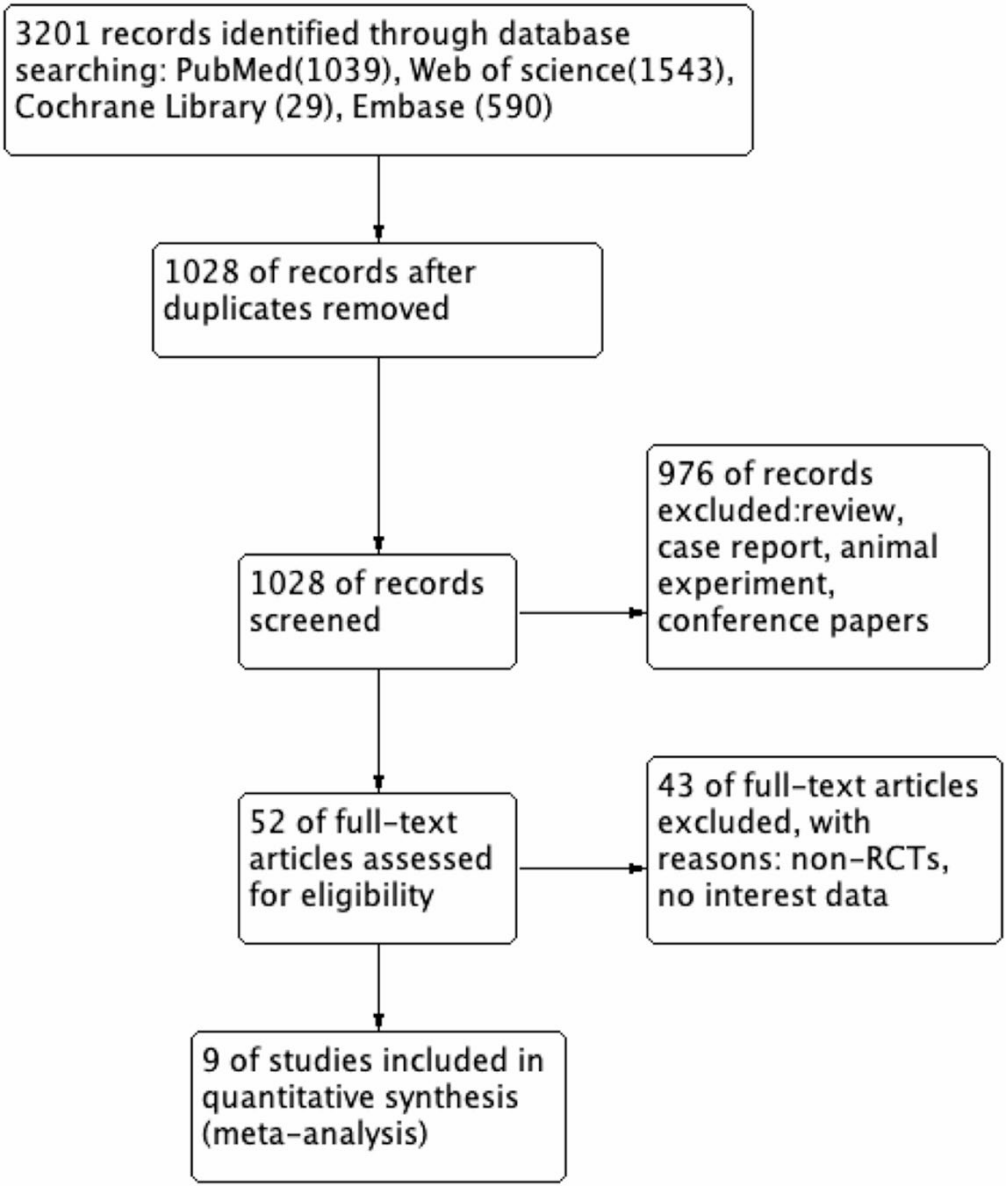
Flow diagram for selected studies in this meta-analysis.

A total of 3201 articles were retrieved in this study. After excluding duplicate articles, review, case reports, and animal experiments, 52 articles were full-text read and screened by reading their titles and abstracts. Finally, 9 articles were included in this meta-analysis^20–28^ (Fig. 1). The basic information of all included studies is shown in Table 1. All the included studies were placebo-controlled studies, involving antioxidants such as coenzyme Q10, selenium, ginger, Nigella sativa, spirulina, Z. multiflora, nettle extract, Resveratrol, and saffron. Among them, 6 studies used capsule formulations, 2 studies used tablet formulations, and 1 study did not specify the formulation. A total of 624 patients with UC were included, with an average age ranging from 34.5 to 41.41 years old. 313 patients received antioxidant supplementation therapy, and 311 patients received placebo-controlled treatment, with a treatment period of 6–12 weeks.

**Table 1:** Baseline characteristics of all included studies in this meta-analysis.

Author, publication year	Type of studies	Sample size		Age		Interventions		Treatment	
		NAS	PB	NAS	PB	NAS	PB	Course	Outcomes
Farnaz, 2021^20^	DB-RCT	43	43	38.39 ± 8.79	40.18 ± 11.46	CoQ10 capsules 200 mg/day	Placebo capsules 200 mg/day	8 weeks	⑤⑥
Khazdouz, 2023^21^	DB-RCT	44	45	34.5 ± 11.2	37.9 ± 10.8	Se capsules (containing 200 µg seleno methionine)	placebo capsules (containing 200 µg rice flour)	10 weeks	⑤⑥
Bodaghi, 2019^22^	DB-RCT	22	24	41.41 ± 11.4	39.21 ± 11.81	Ginger capsules 1000 mg, bid	Placebo capsules 1000 mg, bid	12 weeks	①③⑤⑥
Mehrnaz, 2019^23^	DB-RCT	24	24	34.83 ± 11.2	39.21 ± 11.81	Nigella sativa capsules 500 mg/day	Placebo capsules 500 mg/day	6 weeks	①③⑤⑥
Moradi, 2024^24^	DB-RCT	36	37	37.77 ± 11.67	39.48 ± 11.03	Spirulina capsule 500 mg, twice per day	placebo capsules containing 500 mg corn starch, twice per day	8 weeks	①②③④⑤⑥
Morvaridi, 2025^25^	triple-blind RCT	46	46	38.0 ± 8.6	36.6 ± 11.6	Z. multiflora extract at 6 mg/kg/day	unknown	2 months	①②③⑥
Nematgorgani, 2017^26^	DB-RCT	30	29	36.60 ± 10.89	38.31 ± 13.29	Nettle extract (400 mg per tablet), tid	placebo tablets (400 mg wheat starch per tablet), tid	12 weeks	①⑥
Samsamikor, 2016^27^	DB-RCT	28	28	37.43 ± 16.54	38.78 ± 11.65	Resveratrol capsules 500 mg/day	Placebo capsules 500 mg/day	6 weeks	①②③⑤⑥
Tahvilian, 2021^28^	DB-RCT	40	35	40.55 ± 12.71	40.97 ± 1.34	Saffron tablets 100 mg/daily	Placebo tablets 100 mg/daily	8 weeks	①②③④⑥

DB-RCT, double-blind randomized controlled trial; NAS, natural antioxidant substances; PB, placebo; ① MDA, malondialdehyde; ② SOD, superoxide dismutase; ③ TAC, total antioxidant capacity; ④ GPX, glutathione peroxidase; ⑤SIBDQ, the short IBD questionnaire score; ⑥SCCAI, the simple clinical colitis activity index score.

**Fig. 2:**
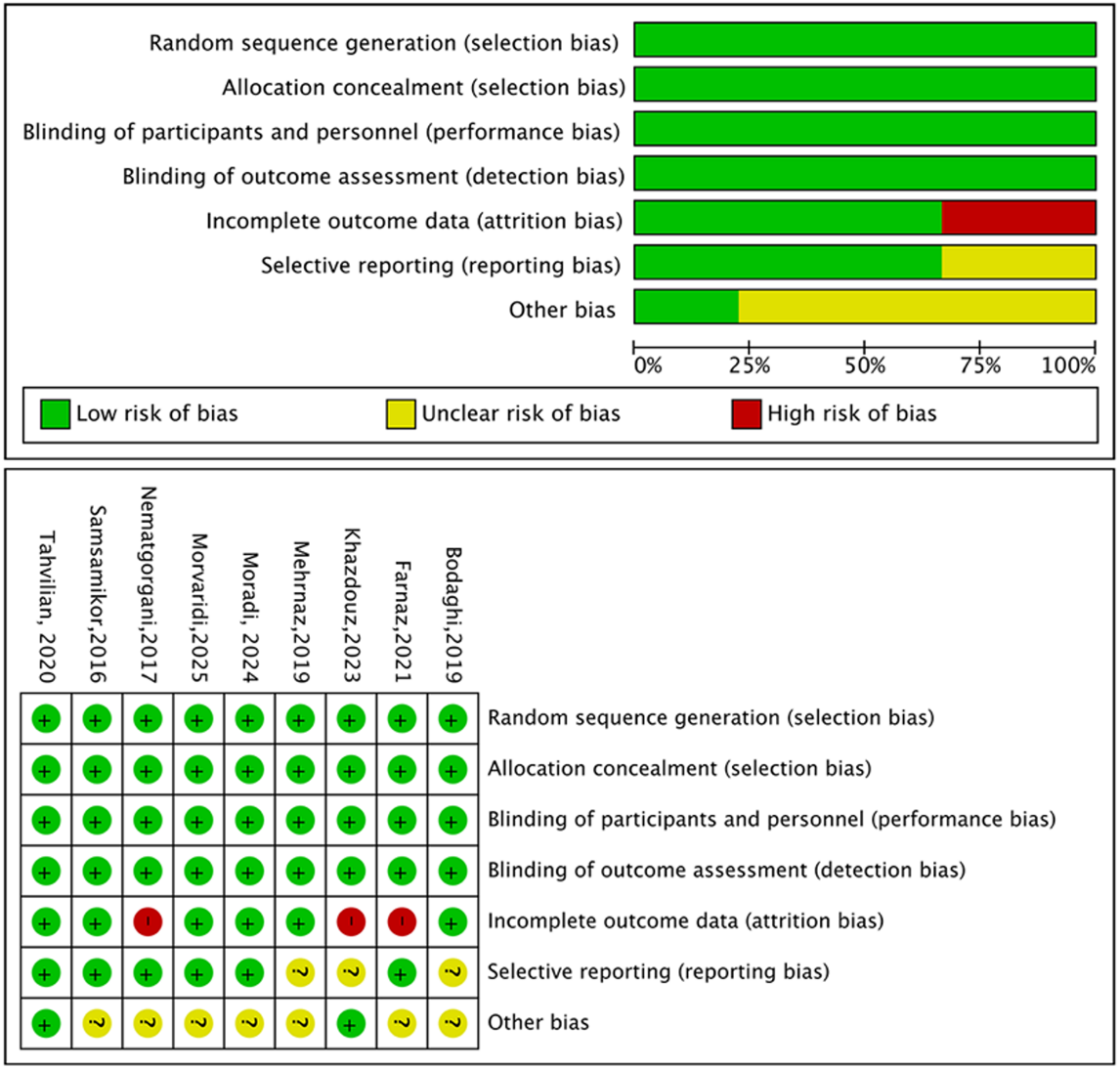
Risk of bias graph and summary. Red (−), high risk of bias; green (+), low risk of bias; yellow(?), unclear risk of bias.

The Cochrane bias risk tool was used to evaluate the quality of 9 studies (Fig. 2). All included studies introduced the methods of generating random sequences, blinding, and described the implementation of allocation concealment and blinding in outcome evaluation, which were rated as low-risk. Three studies have incomplete data and were rated as high-risk. Seven studies did not provide detailed descriptions of other biases and were rated as unknown risk. Overall, the quality of the literature included in this study is relatively high.

### Oxidative stress markers

**Fig. 3:**
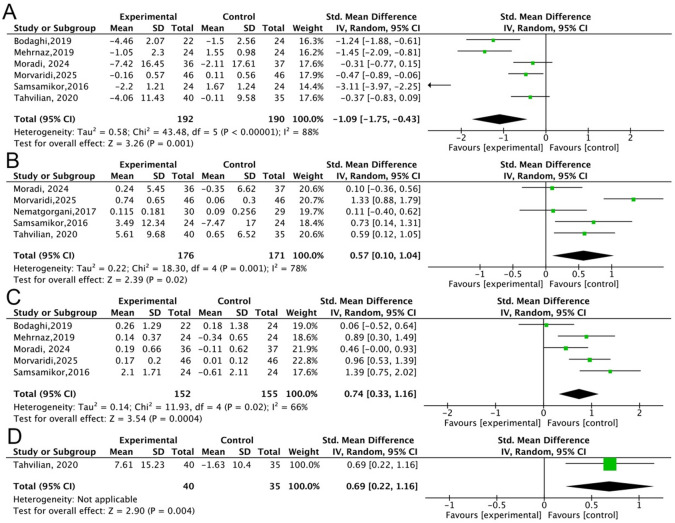
The effect of natural antioxidant substances on the changes in oxidative stress indicators (MDA, SOD, TAC, GPX). (**A**), MDA. (**B**), SOD. (**C**), TAC. (**D**), GPX.

In this study, we evaluated the effect of natural antioxidant substances on the changes in oxidative stress indicators (MDA, SOD, TAC, GPX). Six studies evaluated the effect of antioxidant substances on the change of MDA, and the results showed significant heterogeneity (*P* < 0.00001, *I*^*2*^ = 88%); a random effects model was used for analysis (Fig. 3A). Antioxidant substances supplementation could significantly reduce the level of MDA in patients with UC when compared with placebo (*P* = 0.001, SMD=-1.09, 95%CI=-1.75 to -0.43). Five studies evaluated the effect of antioxidant substances on the change of SOD, TAC, and a random effects model was used due to significant heterogeneity (*P* = 0.001, *I*^*2*^ = 78%; *P* = 0.02, *I*^*2*^ = 66%) (Fig. 3B and C). There is a significant difference in the changes of SOD, TAC between the two groups (*P* = 0.02, SMD = 0.57, 95%CI = 0.10 to 1.04; *P* = 0.0004, SMD = 0.74, 95%CI = 0.33 to 1.16). This result indicates that antioxidant substances supplementation remarkably increased the levels of SOD and TAC in patients with UC. In addition, only one study assessed the effect of antioxidant substances on the change of GPX. Antioxidant substances supplementation could also obviously increase the levels of GPX (*P* = 0.004, SMD = 0.69, 95%CI = 0.22 to 1.16) (Fig. 3D). From these results, we infer that antioxidant substances supplementation can improve the oxidative stress status and enhance the oxidative stress capacity of patients with UC.

### Assessment of disease activity and quality of life

**Fig. 4:**
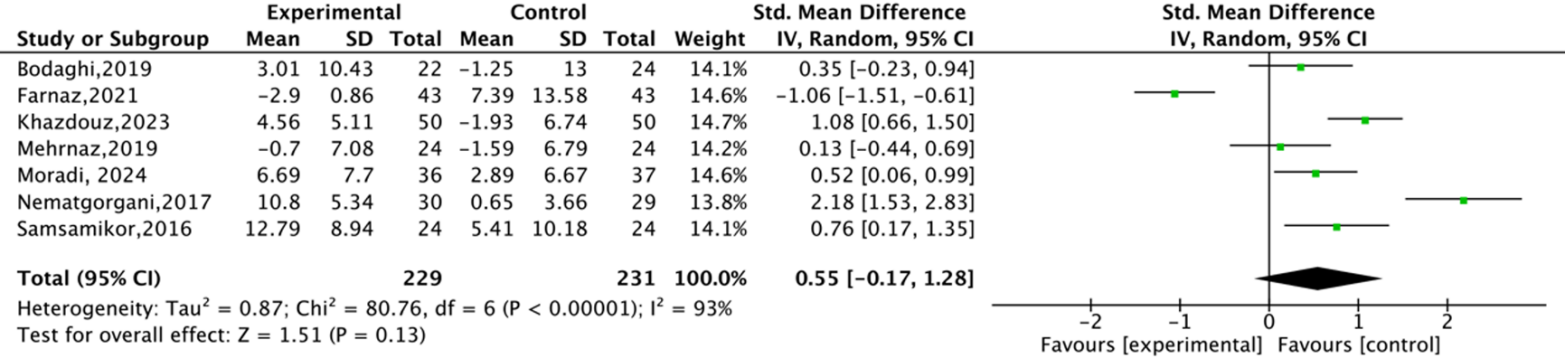
The effects of natural antioxidant substances on SCCAI score.

**Fig. 5:**
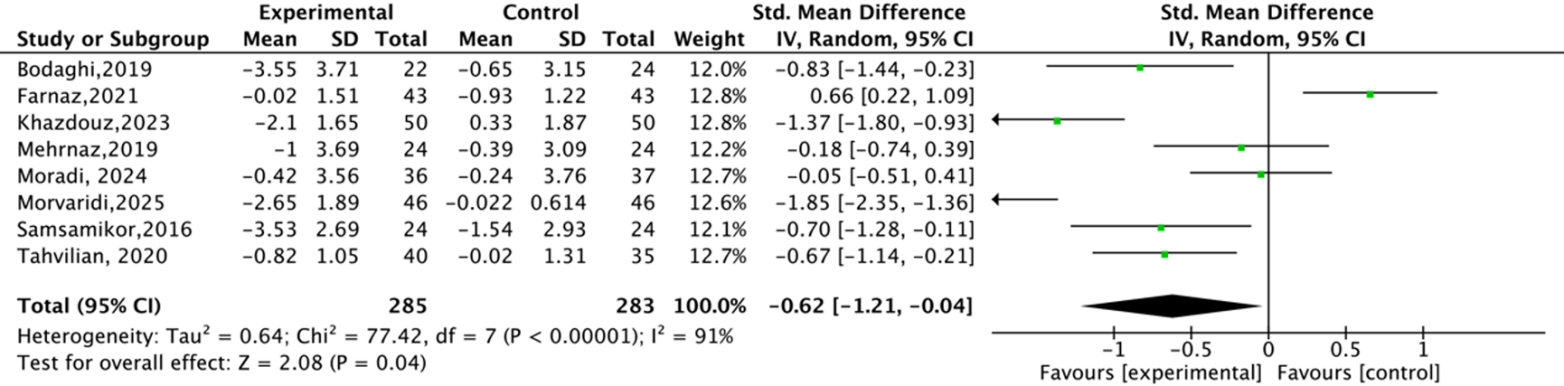
The effects of natural antioxidant substances on IBDQ score in UC patients.

The disease activity of UC patients was assessed using the SCCAI questionnaire, which is a validated questionnaire and closely related to the biochemical parameters of UC. The quality of life of UC patients was evaluated by the IBDQ questionnaire, which contained four aspects: gastrointestinal, systemic, emotional, and social disorders. Eight studies assessed the effect of antioxidant substances on the disease activity of UC patients (Fig. 4). Significant heterogeneity was observed, and a random effects model was used (*P* < 0.00001, *I*^*2*^ = 91%). Antioxidant substances supplementation could significantly increase the change of SCCAI score compared to the placebo group (*P* = 0.04, SMD=-0.62, 95%CI=-1.21 to -0.04). In addition, seven studies assessed the effect of antioxidant substances on the changes in IBDQ score in UC patients (Fig. 5). The result showed that the change of IBDQ score was no obvious difference between groups (*P* = 0.13, SMD = 0.55, 95%CI=-0.17 to 1.28). These results indicated that antioxidant substances supplementation significantly improves the disease activity of UC patients, but had no significant impact on their quality of life.

### Sensitivity analysis and publication bias

**Fig. 6:**
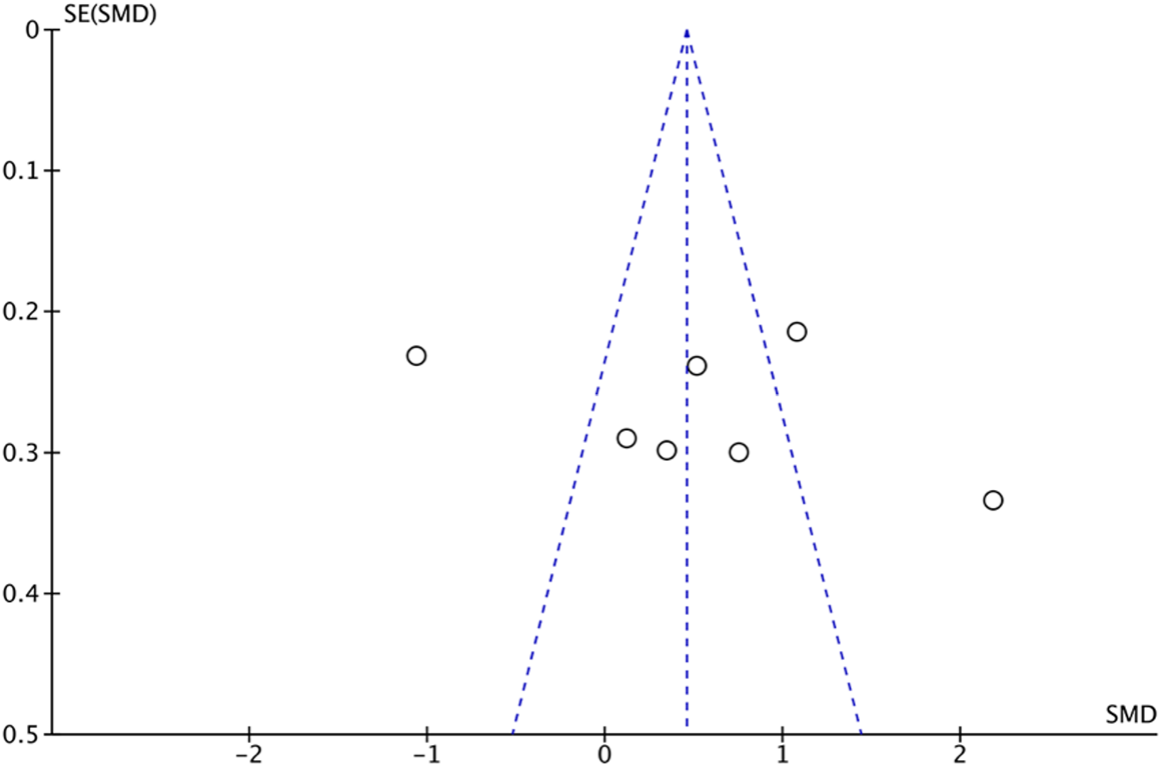
The funnel plot was used to assess the potential publication bias.

During data analysis, sensitivity analysis was used to assess the accuracy of results, but the results did not show any qualitative changes. In addition, the funnel plot is asymmetric and has potential publication bias (Fig. 6). Furthermore, the Eggers test and Beggs test were used to evaluate publication bias. The results showed that *P*_Eggers_=0.014 and *P*_Beggers_=0.036, with *P* values less than 0.05, indicated that potential publication bias existed in this study.

## Discussion

This meta-analysis is the first to comprehensively evaluate the effects of natural antioxidants (coenzyme Q10, selenium, ginger, Nigella sativa, spirulina, Z. multiflora, nettle extract, Resveratrol, saffron) on oxidative stress and clinical outcomes in patients with ulcerative colitis (UC). The results indicate that natural antioxidants supplementation can reduce MDA level and increase the levels of SOD, TAC, and GPX. Moreover, natural antioxidants supplementation significantly decreased the SCCAI scores, but there was no significant difference in IBDQ scores. From these results, we inferred that natural antioxidants supplementation could significantly enhance the antioxidant capacity of UC patients, alleviate oxidative stress states, and reduce disease activity, but had no significant impact on quality of life. These conclusions suggest that antioxidants indeed have significant benefits in the treatment of UC, but their complex mechanisms of action require in-depth analysis in combination with multidimensional evidence.

Oxidative stress is one of the pathogenesis mechanisms of UC. In addition to directly damaging the intestinal tissues, excessive free radicals in the intestinal mucosa of patients with UC can also trigger a chain reaction of lipid peroxidation of polyunsaturated fatty acids on the cell membrane and produce lipid peroxides such as MDA^29^. Both lipid peroxidation reactions and their products can change the stability of the cell membrane and the activity of various enzymes. Moreover, they have chemotactic activity, which can stimulate the production of pro-inflammatory cytokines and continuously amplify the damage of colitis, thereby causing the colonic mucosal damage^30^. NO is another important free radical. Excessive accumulation of NO can block mitochondrial function and hinder DNA synthesis, leading to damage to mucosal cells. It can also combine with superoxide anions and transform into peroxynitrite (OONO -), which is further decomposed into OH and NO_2_ free radicals, causing greater cytotoxicity to the body^31^. SOD is an important antioxidant enzyme system existing in living organisms, which can effectively eliminate oxygen-free radicals and inhibit lipid peroxidation reactions in intestinal tissues^32^. The main function of glutathione peroxidase (GPX) is to eliminate lipid-free radicals, protect biological membranes from free radical damage, and thus play a role in protecting cell structure and function^33^. At the same time, GPX also has the function of eliminating H_2_O_2_. The imbalance between lipid peroxidation reaction and the ability to resist lipid peroxidation in UC patients is an important mechanism causing intestinal mucosal damage^34^.

A systematic review and meta-analysis predicted oxidative stress-related biomarkers in inflammatory bowel diseases (IBD), and found that compared to healthy control patients, active and inactive IBD patients had significantly increased accumulation of oxidative damage biomarkers (MDA), while various antioxidants (GPX/TAC) were decreased^7^. This study found that natural antioxidants supplementation could significantly reduce MDA level and increase the levels of SOD, TAC, and GPX, which was consistent with the results of the included literature. The decrease in MDA levels indicates that lipid peroxidation reactions have been effectively inhibited. Animal experiments have shown that a decrease in MDA levels can reduce apoptosis of colonic epithelial cells and promote mucosal barrier repair^35^. MDA, as a marker of lipid peroxidation, accumulates and leads to the degradation of tight junction proteins (such as ZO-1 and occludin) in the intestinal epithelium, increasing intestinal mucosal permeability^36^. The activity of antioxidant enzymes such as SOD, TAC, and GPX was increased, indicating that the antioxidant defense system was enhanced in the body. Additionally, in all included studies, all the participants did not receive anticoagulants, antibiotics, immunomodulators, corticosteroids, nonsteroidal anti-inflammatory drugs (NSAIDs), anti-tumor necrosis factor (anti-TNF) agents, dietary supplements, or herbal medicines in the past 1–3 months. Therefore, the effects of these drugs on oxidative stress indicators were excluded, which ensured the accuracy of the results of the impact of natural antioxidant supplementation on oxidative stress.

Besides, this study found that natural antioxidants supplementation significantly reduced the SCCAI score of UC patients, thereby improving disease activity. This was consistent with the results of all included literature. However, in the included studies, two studies found that natural antioxidants supplementation significantly reduced IBDQ scores; Five studies have found that natural antioxidants supplementation significantly increases IBDQ scores. This meta-analysis found that there was no significant difference in the changes of IBDQ score between the two groups, indicating that antioxidant supplementation has no significant impact on the quality of life of UC patients. The improvement of clinical symptoms and quality of life in patients with UC is influenced by various factors, such as differences in the sensitivity of assessment tools, the mismatch between intervention duration and cumulative effects, and the mediating role of the microbiota metabolic axis^37^. The SCCAI score mainly relies on objective indicators such as the frequency of defecation, rectal bleeding, and physician evaluation, which directly correspond to the degree of oxidative damage to the intestinal mucosa. The consistency rate between SCCAI reduction of ≥ 2 points and endoscopic improvement reached 83%, confirming its high sensitivity to antioxidant intervention. IBDQ covers multidimensional subjective feelings such as emotional functioning (anxiety/depression) and social roles^38^. The quality of life of UC patients is often regulated by the gut-brain axis, and chronic oxidative stress induces anxiety and fatigue by activating the hypothalamic pituitary adrenal (HPA) axis^39^. In addition, the literature included in this study mostly focuses on short-term interventions of 6 to 12 weeks, but it takes a longer time to improve the quality of life. In general, the clinical symptom relief (such as cessation of diarrhea) can be achieved within 2 weeks. The recovery of psychological and social functions takes more than 3 months, and studies have shown that significant improvement in IBDQ often occurs after 8 weeks of intervention. Although antioxidants can rapidly reduce oxidative damage, the repair of mucosal structure often takes 6–12 weeks, and the IBDQ score is closely related to the degree of mucosal healing. However, UC patients may experience dysbiosis (such as a reduction in bifidobacteria), which may lead to delayed biotransformation and affect the response speed of quality of life-related indicators.

At the same time, this study has certain limitations: Firstly, the sample size was relatively small, and the differences in intervention methods may affect the heterogeneity of results. Secondly, the time points of interventions were inconsistent, and the difference in natural antioxidants (such as types, formulation, and dosage differences) might limit the assessment of long-term curative effect. Thirdly, the differences in detection methods for oxidative stress markers may lead to the heterogeneity of the method. Fourthly, the data on changes in some outcomes obtained through calculations may affect the accuracy of the results. In the included study, both groups of patients received early relief treatment with 5-aminosalicylic acid drugs, immunomodulators, and biologics. Although adjunctive treatments (such as biologics, immunomodulators, corticosteroids) have antioxidant effects, there is currently no data in this area, and further exploration is needed. It may act as a confounding factor, potentially affecting the observation effect of antioxidant supplementation on oxidative stress markers and disease activity. Therefore, some large-scale and standardized designs of RCTs are needed to clarify the optimal treatment cycle and dosage, and evaluate the safety of drugs in the future.

In summary, this study indicates that natural antioxidants effectively correct oxidative stress imbalance in UC patients by reducing MDA levels and increasing the levels of SOD, TAC, GPX, and resulting in a significant improvement in disease activity. However, improving the quality of life requires breaking through the barriers of bioavailability of natural antioxidants, extending intervention cycles, and integrating neuropsychological interventions.

## Data Availability

All the data (used and analyzed) in this study are available from the corresponding author on request.
